# Quantifying longitudinal right ventricular dysfunction in patients with old myocardial infarction by using speckle-tracking strain echocardiography

**DOI:** 10.1186/1476-7120-11-23

**Published:** 2013-06-27

**Authors:** Katsuhisa Konishi, Kaoru Dohi, Muneyoshi Tanimura, Yuichi Sato, Kiyotaka Watanabe, Emiyo Sugiura, Naoto Kumagai, Shiro Nakamori, Hiroshi Nakajima, Tomomi Yamada, Katsuya Onishi, Mashio Nakamura, Tsutomu Nobori, Masaaki Ito

**Affiliations:** 1 Department of Cardiology and Nephrology, Mie University Graduate School of Medicine, 2-174 Edobashi, Tsu 514-8507, Japan; 2 Department of Molecular and Laboratory Medicine, Mie University Graduate School of Medicine, 2-174 Edobashi, Tsu 514-8507, Japan; 3 Department of Translational Medical Science, Mie University Graduate School of Medicine, 2-174 Edobashi, Tsu 514-8507, Japan; 4 Department of Clinical Cardiovascular Research, Mie University Graduate School of Medicine, 2-174 Edobashi, Tsu 514-8507, Japan

**Keywords:** Echocardiography, Right Ventricle, Myocardial Infarction, Plasma Brain Natriuretic Peptide

## Abstract

**Background:**

We investigated longitudinal right ventricular (RV) function assessed using speckle-tracking strain echocardiography in patient with myocardial infarction (MI), and identified the contributing factors for RV dysfunction.

**Methods:**

We retrospectively studied 71 patients with old MI (the OMI group) and 45 normal subjects (the Control group) who underwent a transthoracic echocardiography. Global and free wall RV peak systolic strains (PSSs) in the longitudinal direction were measured by using speckle-tracking strain echocardiography. Left ventricular (LV) PSSs were measured in the longitudinal, radial and circumferential directions. Cardiac hemodynamics including peak systolic pulmonary artery pressure was also assessed non-invasively. Plasma brain natriuretic peptide (BNP) levels were measured in all patients.

**Results:**

In the OMI group, 73% of the patients had a normal estimated peak systolic pulmonary artery pressure of less than 35 mmHg. Global and free wall RV PSS were impaired in the OMI group compared with the Control group, and these RV systolic indices were significantly associated with heart rate, logarithmic transformed plasma BNP, greater than 1 year after onset of MI, Doppler-derived estimated pulmonary vascular resistance, LV systolic indices, LV mass index, infarcted segments within a territory of the left circumflex artery and residual total occlusion in the culprit right coronary artery. Multivariable linear regression analysis indicated that reduced longitudinal LV PSS in the 4-chamber view and BNP levels ≥500 pg/ml were independently associated with reduced global and free wall RV PSS. Moreover, when patients were divided into 3 groups according to plasma BNP levels (BNP <100 pg/ml; n = 31, 100 ≤BNP <500 pg/ml; n = 24, and BNP ≥500 pg/ml; n = 16), only patients with BNP ≥500 pg/ml had a strong correlation between RV PSS and longitudinal LV PSS in the 4-chamber view (r = 0.78 for global RV PSS and r = 0.71 for free wall RV PSS, p <0.05).

**Conclusion:**

Longitudinal RV systolic strain depends significantly on longitudinal LV systolic strain especially in patients with high plasma BNP levels, but not on estimated peak systolic pulmonary artery pressure. These results indicate that process of RV myocardial dysfunction following MI may be governed by neurohormonal activation which causing ventricular remodeling rather than increased RV afterload.

## Introduction

Myocardial infarction (MI) is associated with compensatory mechanisms involving both the left and right ventricles, and, even if the right ventricle is initially spared, right ventricular (RV) structure and function can still be altered later on [1]. Importantly, RV function is a known predictor of cardiovascular morbidity and mortality in patients with LV dysfunction after MI [2]. However, since myocardial function and structure are chronically modulated by a complex interplay of multiple factors, such as hemodynamic load and neurohumoral stimulation, the mechanisms leading to RV dysfunction following MI are not completely investigated in the clinical setting [3,4]. Furthermore, echocardiographic evaluation of RV function has been more challenging than that of left ventricle mainly because of the complex structure and asymmetrical shape of the right ventricle. The recent introduction of speckle-tracking echocardiography provides objective measures to quantify segmental and global ventricular function independently of angle of incidence, chamber translation, cardiac rotation, and ventricular size [5,6]. Accordingly, we investigated longitudinal RV function assessed using speckle-tracking strain echocardiography in patient with old MI (OMI), and identified the contributing factors for RV dysfunction.

## Patients and methods

### Study population

We retrospectively reviewed the consecutive echocardiographic data between July 2005 and January 2009, and selected 71 patients with OMI (the OMI group: 67 ± 11 years) who underwent complete echocardiography including optimal RV-focused apical four-chamber images using Vivid 7 among various echocardiography ultrasound systems in our echo laboratory at Mie University Hospital, which were available for off-line analysis (GE-Vingmed Ultrasound AS, Horten, Norway). All patients had experienced MI for over 1month and were diagnosed with reference to their personal history, physical examinations, laboratory tests, electrocardiography, echocardiography, coronary angiography, and cardiac magnetic resonance imaging. Patients were excluded from the study if they had suboptimal images, atrial fibrillation, significant primary valvular heart disease, prosthetic valves, or a pacer wire in the right ventricle. To minimize the influence of confounding factors in the evaluation of RV function after MI in the LV myocardium, patients with a coexisting RV infarction were excluded in the present study. Patients who had been diagnosed with non-ischemic dilated cardiomyopathy before the onset of MI were also excluded in the present study. The diagnosis of RV infarction was defined by ST segment elevation >0.1 mV in lead V4R in the 12-lead electrocardiography examination. Non-existence of obvious RV infarction was confirmed by using cardiac MRI and coronary angiography in patients with silent OMI who did not undergo 12-lead electrocardiography examination at the onset of MI. We also studied 45 age-matched and gender-matched normal subjects (the Control group: 66 ± 11 years) who had no history of cardiopulmonary disease, and who had normal electrocardiographic results and normal echocardiographic results. The protocol was approved for use by the Human Studies Subcommittee of Mie University’s Graduate School of Medicine.

### Echocardiography

Arm-cuff blood pressure measurements were performed at the beginning of the echocardiographic study for all subjects [6]. Interventricular septal and LV posterior wall thickness, LV end-diastolic dimension, end-systolic dimension, and fractional shortening were assessed from the parasternal long axis view. LV volume indices and ejection fraction were assessed using biplane Simpson’s rule. LV mass index was calculated on the basis of the area-length method [6]. The Doppler-derived stroke volume was normalized by body surface area. Ratio of peak early to late diastolic transmitral flow velocity (mitral E/A) and the deceleration time (DT) of E velocity was calculated using pulsed Doppler echocardiography [6,7]. Averaged peak early diastolic mitral annular velocity (Ea) at the inferior-septal and LV lateral site was used as a marker of LV diastolic function. The E/Ea ratio was calculated as a Doppler parameter reflecting LV filling pressure [8]. The RV end-diastolic area (EDA) index, the end-systolic area (ESA) index, and the fractional area change (FAC) from the apical 4-chamber view were also measured [5]. Tricuspid annular plane systolic excursion (TAPSE) was measured for assessing longitudinal RV systolic function [9]. The RV myocardial performance index (MPI) was calculated as the sum of the isovolumic contraction time and isovolumic relaxation time divided by the ejection time [5,9,10]. Estimated peak systolic pulmonary artery pressure was calculated from the sum of the maximal pressure difference between the right ventricle and the right atrium, as calculated by the continuous-wave Doppler flow velocity, and the mean right atrial pressure, as estimated by the diameter of the inferior vena cava and its respiratory variation [9]. Pulmonary vascular resistance (PVR) was noninvasively estimated using echocardiography [11]. All echocardiographic measurements represent the average of 3 beats. Blood test including measurement of plasma brain natriuretic peptide (BNP) levels was performed within the 2 weeks prior to or after the echocardiography examination.

### Speckle-tracking strain and displacement analysis

**Figure 1:**
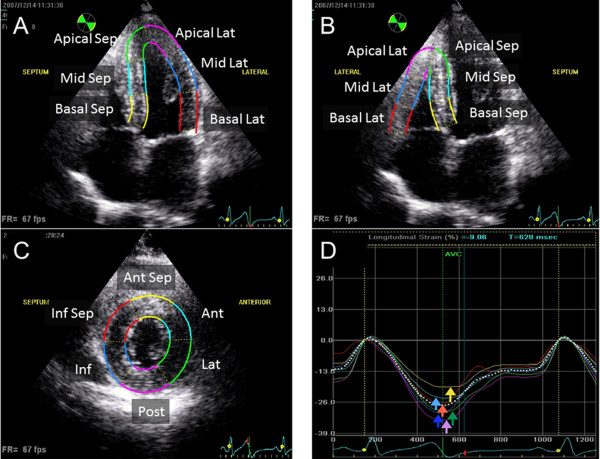
**Six-segmental models were created using a tracking algorithm after manual delineation of the endocardial border in the LV apical 4-chamber view (A) and RV apical 4-chamber view (B), and the short axis view for the left ventricular circumferential and radial functional measurements (C).** Right bottom panel shows time-strain curves RV apical 4-chamber view for the assessment of longitudinal myocardial systolic function. Solid colored lines indicate corresponding segmental strain curves, and the white dotted line indicates global strain curves **(D)**.

Speckle-tracking analysis was used to generate regional and global myocardial strain in both the left and right ventricles (Figure 1). Longitudinal LV strain was assessed in the apical 4-chamber, 2-chamber, and long axis views, and radial and circumferential LV strains were assessed in the parasternal short-axis views at the mid-LV level. Longitudinal RV strain was assessed in the apical 4-chamber view [12]. Average frame rate for the analysis was 64 ± 9 Hz. For speckle-tracking echocardiography assessment, routine B-mode gray scale images were analyzed using commercially available software (EchoPAC, GE Vingmed, Horton, Norway). Myocardial strain is expressed as the percent change from the original dimension at end-diastole, and myocardial thickening or lengthening was represented as a positive value, while myocardial thinning or shortening was represented as a negative value [5,6]. The software automatically divided into 6 standard segments in the LV apical 4-chamber, 2-chamber, long-axis, mid-LV short-axis, and RV apical 4-chamber views, respectively (Figure 1). Peak systolic strain (PSS) obtained from time-strain curves were defined as the indices of myocardial systolic contraction. Global RV PSS in the 6 segments was assessed, and the free wall RV PSS was obtained by averaging 3-site strain signals simultaneously (the basal RV lateral wall, the mid RV lateral wall, and the apical RV wall). Global LV radial PSS was obtained by averaging 6-site strain signals simultaneously [13].

### Clinical outcomes

After echocardiography assessments, patients were followed for 4.7 ± 2.3 years (median 5.3 years). The study end-point was heart failure hospitalization or cardiovascular death. The causes of death were determined by the attending doctors blinded to the group assignment.

### Statistical analysis

The mean of each continuous variable was presented along with the standard deviation. Categorical variables were presented as percent frequencies. Between-group comparisons were assessed using analysis of variance for continuous variables and the Fisher exact test for categorical data. Bonferroni’s correction was applied for multiple comparisons. Associations between RV PSS and independent variables were examined through Pearson’s correlation coefficient and linear regression. Multivariate linear regression analyses were performed to examine the independent correlates between RV PSS and clinical, echocardiographic and laboratory parameters. The β-coefficients were standardized regression coefficients. Logarithmic transformation was performed to achieve approximate normal distribution for BNP. Intra-observer variability was determined by having one observer repeat the measurement of global RV PSS and free wall RV PSS d observer measure these variables in the same datasets. Intra- and inter-observer variability values were calculated as the absolute difference between the corresponding two measurements as a percentage of the mean. *P* values < 0.05 were considered statistically significant. Analyses were performed using SPSS for Windows, version 19 (SPSS, Inc, Chicago, IL).

## Results

### Clinical and echocardiographic characteristics

There were a total of 99 culprit territory vessels. Forty-six patients (65%) had one infarcted segment within a territory of the left anterior descending artery (n = 29), the left circumflex artery (n = 8), or the right coronary artery (n = 9), and 25 patients (35%) had more than one infarcted segments. The time interval from the first onset of MI was <1 year in 28% of patients, ≥1 year (range 1–35 years) in 39% of patients, and was unknown in 32% of patients mainly because of silent MI. Successful primary revascularization early in the course of AMI was achieved only in 27 patients and 27% of the all culprit coronary lesions responsible for MI, mainly due to high prevalence of patients with silent ischemia with multivessel disease. There were 26 vessels with residual total occlusion in the culprit lesion responsible for MI. Table 1 shows the clinical characteristics of the study groups. There were no statistical differences in height, body mass index, blood pressure, and heart rate between the Control and OMI group. Estimated glomerular filtration rate [14] was lower in the OMI group compared to the Control group. Plasma BNP levels were varied from 2 to 3000 pg/ml with median value of 162 pg/ml in the OMI group.

**Table 1:** Clinical characteristics of the study subjects

	**The control group (n = 45)**	**The OMI group (n = 71)**
Demographics		
Mean age (years)	66 ± 11	67 ± 11
Male gender (%)	78	85
Height (cm)	162 ± 10	162 ± 9
Body mass index (kg/m^2^)	23 ± 3	23 ± 3
SBP (mmHg)	123 ± 13	118 ± 23
Heart rate (beats/min)	65 ± 8	68 ± 12
Medical history		
Hypertension (%)	0	63*
Diabetes (%)	0	45*
Dyslipidemia (%)	0	63*
Current smoking (%)	2	23*
Obesity (%)	0	1
Medication use		
Beta blocker (%)	0	56*
Calcium channel blocker (%)	0	17*
ACEI/ARB (%)	0	89*
Aldosterone blocker (%)	0	31*
Diuretic (%)	0	41*
Measurements		
Hemoglobin (g/dl)	12.9 ± 1.9	12.3 ± 2.0
eGFR (ml/min/1.73 m2)	69 ± 19	53 ± 20*
BNP (pg/ml)	-	343 ± 519

*OMI* old myocardial infarction, *SBP* systolic blood pressure, *DBP* diastolic blood pressure, *ACEI* angiotensin converting enzyme inhibitor, *ARB* angiotensin receptor blocker, *eGFR* estimated glomerular filtration rate, *BNP* brain natriuretic peptide. *P <.05 vs. the Control group.

Table 2 shows the left-sided echocardiographic data of the study subjects. The OMI group had thickened LV wall, large LV chamber size, greater LV mass index, and reduced LV ejection fraction compared with the Control group, indicating dilatational LV remodeling in patients with OMI. Although mitral E/A and DT of the E velocity were similar in the 2 groups, the OMI group had lower Ea and higher E/Ea compared with the Control group. Table 3 shows the right-sided echocardiographic data of the study subjects. Although RV area indices and FAC were similar in both groups, TAPSE was reduced and RV MPI was higher in the OMI group compared with the Control group. In the OMI group, 73% of patients had normal estimated peak systolic pulmonary artery pressure of less than 35 mmHg.

**Table 2:** Left-sided echocardiographic data of the study subjects

	**The control group (n = 45)**	**The OMI group (n = 74)**
IVST (mm)	10 ± 1	11 ± 2*
PWT (mm)	10 ± 1	11 ± 2*
LV Dd (mm)	44 ± 4	51 ± 10*
LV Ds (mm)	27 ± 5	38 ± 12*
LV FS (%)	41 ± 5	26 ± 12*
EDV index (ml/m^2^)	38 ± 7	61 ± 27*
ESV index (ml/m^2^)	13 ± 4	36 ± 24*
LV EF (%)	65 ± 6	45 ± 16*
LV stroke volume (ml)	58 ± 11	54 ± 15
LV mass index (g/m^2^)	84 ± 15	151 ± 55*
E/A	1.0 ± 0.2	1.0 ± 0.6
DT (msec)	230 ± 65	220 ± 77
Ea (cm/s)	6.8 ± 1.7	4.8 ± 2.4*
E/Ea	9.5 ± 3.3	17.7 ± 10.4*

*OMI* old myocardial infarction, *IVST* interventricular septal thickness, *PWT* posterior wall thickness, *LV* left ventricular, *Dd* end-diastolic dimension, *Ds* end-systolic dimension, *FS* fractional shortening, *EDV* end-diastolic volume, *ESV* end-systolic volume, *EF* ejection fraction, *E/A* ratio of peak early to late diastolic transmitral flow velocity, *DT* deceleration time of the mitral peak early diastolic transmitral flow velocity, *Ea* peak early diastolic mitral annular velocity, *E/Ea* ratio of peak early diastolic transmitral flow velocity to Ea. *P <.05 vs. the Control group.

**Table 3:** Right-sided echocardiographic data of the study subjects

	**The control group (n = 45)**	**The OMI group (n = 74)**
RV EDA index (cm^2^/m^2^)	8 ± 3	8 ± 2
RV ESA index (cm^2^/m^2^)	4 ± 2	4 ± 2
RV FAC (%)	53 ± 9	51 ± 11
TAPSE (mm)	19 ± 4	16 ± 5*
RV MPI	0.30 ± 0.10	0.39 ± 0.21*
Peak PA pressure (mm Hg)	-	31 ± 15
PVR (Wood units)	-	1.8 ± 0.8

*OMI* old myocardial infarction, *RV* right ventricular, *EDA* end-diastolic area, *ESA* end-systolic area, *FAC* fractional area change, *TAPSE* tricuspid annular plane systolic excursion, *MPI* myocardial performance index, *PA* pulmonary artery, *PVR* pulmonary vascular resistance. *P <.05 vs. the Control group.

### Strain measurements

**Figure 2:**
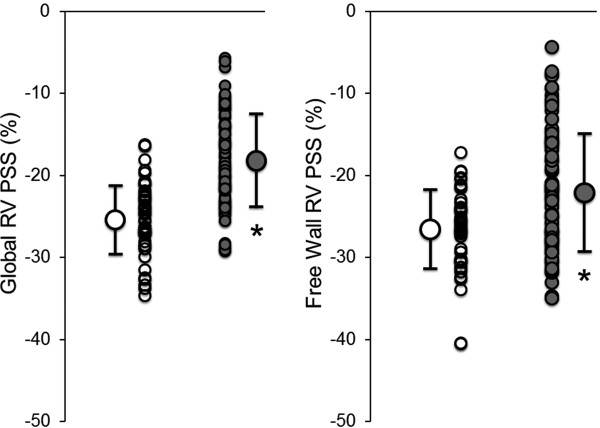
**Plots shows comparisons of global (left) and free wall (right) RV peak systolic strain (PSS) between the Control (open circle, n = 45) and OMI groups (gray circle, n = 71).** *p <0.05 vs. the Control group.

Speckle tracking was possible in 100% of the 3480 attempted segments from the 116 echocardiographic studies with technically adequate images. Table 4 and Figure 2 show comparisons of LV and RV strains between the Control and OMI groups. All strain values in the left ventricle were significantly reduced in the OMI group compared with those in the Control group. Notably, both global and free wall RV PSS were significantly reduced in the OMI group compared with those in the Control group (global RV PSS: -18.3 ± 5.9* vs. -25.5 ± 4.2%, and free wall RV PSS: -22.1 ± 7.5* vs. -26.9 ± 5.0%, *p <0.05 vs. the Control group, Figure 2).

**Table 4:** Left and right ventricular strain

	**The control group (n = 45)**	**The OMI group (n = 74)**
LV PSS (L: 4-chamber) (%)	−17.6 ± 3.1	−12.0 ± 5.1*
LV PSS (L: 2-chamber) (%)	−18.8 ± 3.8	−12.2 ± 5.5*
LV PSS (L: long axis) (%)	−17.3 ± 3.5	−11.3 ± 4.7*
LV PSS (R) (%)	52.1 ± 15.0	31.6 ± 22.8*
LV PSS (C) (%)	−20.4 ± 3.6	−12.3 ± 5.5*

*OMI* old myocardial infarction, *LV* left ventricular; *PSS* peak systolic strain, *L* longitudinal, *R* radial, *C* circumferential. *P <.05 vs. the Control group.

### Determinants of RV strain

Successful primary revascularization was not statistically associated with global or free wall RV PSS. We assessed the potential impact of infarcted site and residual total occlusion in the culprit lesion responsible for MI on RV function. When patients were divided into two groups according to median value of the global RV PSS: -19.1%, patients with reduced global RV PSS (−13 ± 4%) had a higher prevalence of residual total occlusion in the culprit right coronary artery than those with preserved global RV PSS (−23 ± 3%) (Table 5). There were 31 patients (44%) who received beta blockers. No statistically significant differences were observed in the values of global and free wall RV PSS between patients with and without beta-blocker therapy.

**Table 5:** Comparison of clinical and echocardiographic data between patients with preserved and reduced free-wall RV PSS

	**Reduced global RV PSS (n = 35)**	**Preserved global RV PSS (n = 36)**
Culprit lesions responsible for MI		
Single LAD (n)	11	18
Single LC (n)	6	2
Single RCA (n)	3	6
Multi-vessels (n)	15	10
Residual total occlusion of the culprit lesions		
LAD (n)	3	7
LC (n)	3	1
RCA (n)	10	2*

*RV* right ventricular, *PSS* peak systolic strain, *MI* myocardial infarction, *LAD* left anterior descending artery, *LC* left circumflex artery, *RCA* right coronary artery. *P <.05 vs. patients with reduced global RV PSS.

Since the time interval between the acute MI onset and the echocardiographic evaluation varied individually in the present study, we investigated the potential influence of the chronicity of MI on RV function. Patients <1 year after onset of MI had much more impaired global and free wall RV PSS than those ≥1 year after onset of MI among the 3 groups (global RV PSS: -21 ± 6% in patients <1 year, -17 ± 6*% in patients ≥1 year, and −18 ± 6% in patients with unknown duration, and free wall RV PSS: -25 ± 7% in patients <1 year, -20 ± 8*% in patients >1year, and −22 ± 7% in patients with unknown duration *p<0.05 vs. patients <1 year) although age, gender, log-transformed plasma BNP, and longitudinal LV PSS in the 4-chamber view were similar.

**Figure 3:**
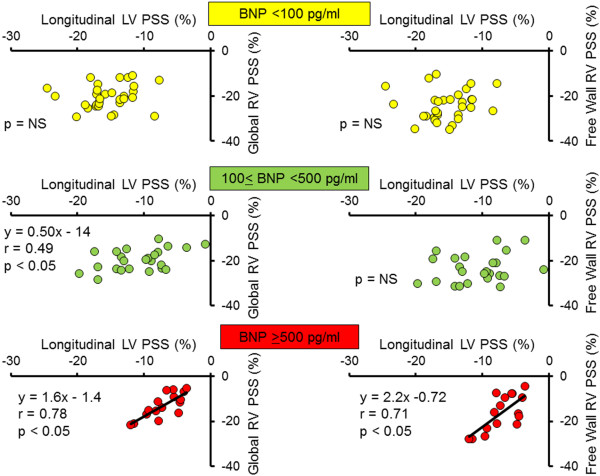
Scatter plots depicting the correlation between global RV PSS and global LV PSS in the apical 4-chamber view (left) and between free wall RV PSS and global LV PSS in the apical 4-chamber view (right) in patient with BNP <100 pg/ml (top, n = 31), 100 ≤BNP <500 pg/ml (middle, n = 24), and BNP ≥500 pg/ml (bottom, n = 16).

**Figure 4:**
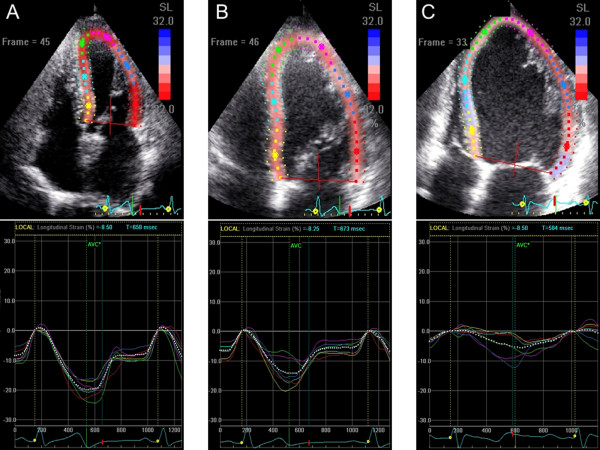
Examples of LV strain imaging in the apical four-chamber view (top) and corresponding time-strain curves (bottom) from a normal subject (A), a patient with OMI in the territory of left anterior descending artery and low plasma BNP level (B), and a patient with OMI in the all 3 coronary artery territories and high plasma BNP level (C).

**Figure 5:**
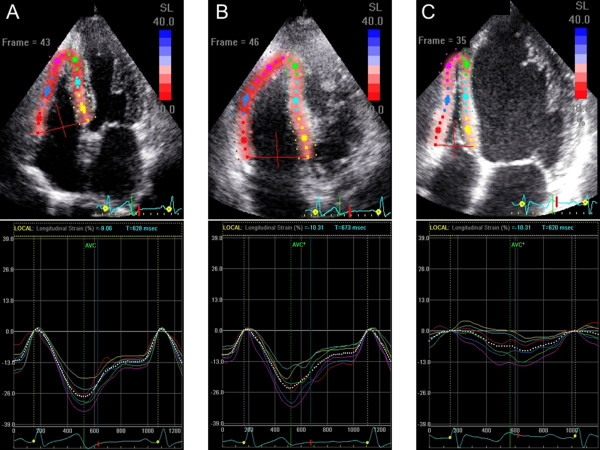
**Examples of RV strain imaging in the apical four-chamber view (top) and corresponding time-strain curves (bottom) from the same subjects shown in the Figure**4**. (A)** the normal subject, **(B)** the patient with OMI in the territory of left anterior descending artery and low plasma BNP level, and **(C)** the patient with OMI in the all 3 coronary artery territories and high plasma BNP level.

Tables 6 and 7 show the univariate correlation coefficients for clinical characteristics and echocardiography variables with global and free wall RV PSS, and the results of the stepwise multivariate regression analyses in the OMI group. Both global and free wall RV PSS were associated with heart rate, plasma BNP levels, chronicity of MI, LV mass index, PVR, LV PSS, and infarcted territories and the presence of residual total occlusion. Multivariable linear regression analysis indicated that longitudinal LV PSS in the 4-chamber view, BNP ≥500 pg/ml, and residual total occlusion in the culprit right coronary artery were the independent determinants of global RV strain. Longitudinal LV PSS in the 4-chamber view, BNP ≥500 pg/ml, MI in the territory of the left circumflex artery were the independent determinants of free wall RV PSS. When patients were divided into 3 groups according to plasma BNP levels (BNP <100 pg/ml; n = 31, 100 ≤BNP <500 pg/ml; n = 24, and BNP ≥500 pg/ml; n = 16), only patients with BNP ≥500 pg/ml had a strong correlation between RV strains and longitudinal LV PSS in the 4-chamber view (Figure 3) although global RV strain had modest correlation with longitudinal LV PSS patients with 100 ≤BNP <500 pg/ml. Figures 4 and 5 and Additional files 1, 2, 3, 4, 5, 6: Movies1-6 show typical examples of strain imaging (top) in the LV apical 4-chamber views (Figure 4) and RV apical 4-chamber views (Figure 5), and corresponding time-strain curves (bottom) in a normal subject, a patient with OMI and low plasma BNP level, and a patient with OMI and high plasma BNP level. The normal subject had preserved LV and RV regional strains through a cardiac cycle (Figures 4A and 5A, and Additional files 1 and 2: Movies for the LV and RV strain images, respectively). The patient with OMI in the territory of left anterior descending artery and low plasma BNP level (39 pg/ml) had mildly reduced segmental longitudinal LV strain in both the apical and apical anterolateral segments and normal free wall RV strain (Figures 4B and 5B, and Additional files 3 and 4: Movies for the LV and RV strain images, respectively). The patient with OMI in the all 3 coronary artery territories and high plasma BNP level (955 pg/ml) had severely reduced LV and RV strain (Figures 4C and 5C, and Additional files 5 and 6: Movies for the LV and RV strain images, respectively). Notably, this patient had non-dilated RV, normal FAC, and normal estimated peak systolic pulmonary artery pressure of 25 mmHg.

**Table 6:** Univariate and multivariate linear regression analysis of variables associated with global RV PSS

	**Univariate**		**Multivariate**	
	**Coefficients**	**p Value**	**β-coefficients**	**p Value**
Heart rate	0.29	0.02	-	ns
Log BNP	0.43	<0.01	-	ns
BNP ≥100 pg/ml	0.27	0.02	-	ns
BNP ≥500 pg/ml	0.47	<0.01	0.25	0.02
MI ≥1 year	0.26	0.03	-	ns
PVR	0.39	<0.01	-	ns
LV mass index	0.29	0.01	-	ns
LV PSS (L: 4-chamber)	0.54	<0.01	0.30	<0.01
Infarction in the LAD territory	−0.06	0.61		
Infarction in the LC territory	0.30	0.01	-	ns
Infarction in the RCA territory	0.19	0.12		
Multi-vessel infarction	0.24	0.04	-	ns
Residual total occlusion in the culprit LAD	−0.03	0.81	-	ns
Residual total occlusion in the culprit LC	0.18	0.13		
Residual total occlusion in the culprit RCA	0.41	<0.01	0.26	0.01

*RV* right ventricular, *PSS* peak systolic strain, *BNP* brain natriuretic peptide, *MI* myocardial infarction, *LV*, left ventricular, *L* longitudinal, *LAD* left anterior descending artery, *LC* left circumflex artery, *RCA* right coronary artery.

**Table 7:** Univariate and multivariate linear regression analysis of variables associated with free wall RV PSS

	**Univariate**		**Multivariate**	
	**Coefficients**	**p Value**	**β-coefficients**	**p Value**
Heart rate	0.24	0.04	-	ns
Log BNP	0.38	<0.01	-	ns
BNP ≥100 pg/ml	0.23	0.06	-	ns
BNP ≥500 pg/ml	0.40	<0.01	0.28	0.02
MI ≥1 year	0.23	0.06	-	ns
PVR	0.30	0.01	-	ns
LV mass index	0.28	0.02	-	ns
LV PSS (L: 4-chamber)	0.45	<0.01	0.26	0.03
Infarction in the LAD territory	−0.11	0.37		
Infarction in the LC territory	0.31	<0.01	0.26	0.01
Infarction in the RCA territory	0.13	0.29		
Multi-vessel infarction	0.13	0.29		
Residual total occlusion in the culprit LAD	−0.11	0.37		
Residual total occlusion in the culprit LC	0.13	0.28		
Residual total occlusion in the culprit RCA	0.36	<0.01	-	ns

*RV* right ventricular, *PSS* peak systolic strain, *BNP* brain natriuretic peptide, *MI* myocardial infarction, *LV* left ventricular, *L* longitudinal, *LC* left circumflex artery, *RCA* right coronary artery.

### Kaplan-Meier analysis

**Figure 6:**
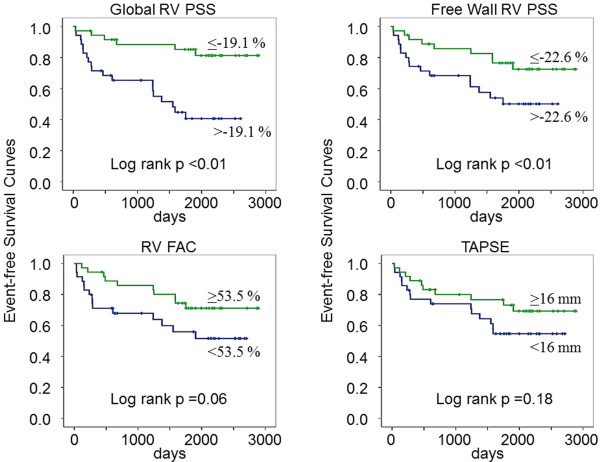
**Kaplan–Meier event-free survival curves for the composite endpoint of heart failure hospitalization and cardiovascular death.** Patients are stratified by global RV PSS (left top), free wall RV PSS (right top), RV FAC (left bottom), and TAPSE (right bottom).

Kaplan–Meier event-free survival curves for the composite endpoint of heart failure hospitalization and cardiovascular death were constructed, and statistical differences between patients with preserved and reduced indices of RV function were assessed by the Log-rank test. During the follow-up periods, there were 6 events (17%) in patients with preserved global RV PSS above the median value, and 19 events (54%) in those with reduced global RV PSS. As shown in Figure 6, patients with reduced global RV PSS and those with reduced free wall RV PSS had a higher risk for composite heart failure hospitalization and cardiovascular death whereas conventional indices of RV function including RV FAC and TAPSE did not reach statistical significance probably because of small sample size.

Inter-observer and intra-observer variability were 5.7 ± 4.4 and 3.6 ± 3.8% for global RV PSS and 5.4 ± 3.6 and 3.8 ± 3.7% for free wall RV PSS.

## Discussion

The major findings of our study include the following: 1) longitudinal RV strain was reduced in patients with OMI in the absence of RV infarction, and 2) RV strain depends highly on longitudinal LV strain especially in patients with high plasma BNP levels. Although LV dysfunction is considered a major mechanism underlying the development of heart failure, several studies have shown the pivotal importance of RV function. Recently, several experimental and clinical studies have demonstrated that longitudinal function mainly reflects global RV systolic function [5,15,16] and is associated with patient outcome [17,18].

The mechanisms leading to RV dysfunction following MI in the LV myocardium are not completely clear [19], but it is frequently assumed that LV failure causes pulmonary hypertension and increased RV afterload leading to RV remodeling and dysfunction. However, the results obtained in the present study suggest that post-infarction RV dysfunction is not solely governed by RV afterload. Optimal medication therapy including diuretics successfully prevented pulmonary hypertension, enlargement of the right ventricle and reduction of FAC in the majority of the study population. Nevertheless, both longitudinal RV strain and TAPSE were reduced in patients with OMI, as shown in Additional file 6: Movie. Toldo et al. assessed changes in LV and RV dimensions and function and their association with the presence and degree of pulmonary hypertension 1 week following experimental acute MI involving the LV free wall in 10 mice [20]. They found that RV FAC and TAPSE declined by 33% and 28% respectively; however, invasively measured RV systolic pressure was within the normal values and unchanged following acute MI. Therefore, they concluded that RV dysfunction develops independent of changes in RV afterload.

Infarction or ischemia of the RV and/or the septum are common in patients with MI and can also contribute to abnormal RV systolic function. Residual total occlusion in the right coronary artery was one of the independent contributors of reduced global RV PSS in the present study, indicating that residual ischemia in the right coronary artery may affect RV function. Contraction of the interventricular septum contributes to RV ejection [21] and efficient RV performance is determined by the proper functional activity of the free wall and the interventricular septum [22]; therefore, RV functional deterioration may progress in part through infarcted myocardium in the septal segments that are partially supplied by the right coronary artery. The present study demonstrated that MI in the territory of the left circumflex artery negatively affects longitudinal free wall RV systolic function. The posterolateral branches of the left circumflex artery supply a portion of the posterior RV free wall in <10% of hearts [9]. Therefore, LV infarction in the territory of left circumflex artery may cause occult RV infarction. Nevertheless, our study demonstrated that systolic strain in the RV free wall segments reduced significantly and was independently associated with longitudinal LV function as assessed by using speckle-tracking strain echocardiography.

Other potential mechanisms underlying RV free wall dysfunction include neurohumoral activation or inflammation [23]. We found that plasma BNP level is associated with longitudinal RV dysfunction. Interestingly, a strong correlation between LV and RV function was observed only in patients having plasma BNP levels above 500 pg/ml. BNP is synthesized predominantly in the left ventricle as a reaction to cardiac wall distension and stretching, and elevated plasma BNP level correlated directly with NYHA score, intra-ventricular pressure, pulmonary pressure, and prognosis [24]. Shah et al. reported that BNP levels >500 pg/ml at discharge was a stronger predictor of mortality in patients hospitalized with acute HF [25]. Vogelsang et al. demonstrated a negative correlation between RV systolic function and BNP levels in patients affected with post-ischemic cardiomyopathy [26] and suggested that circulating BNP might be synthesized in the right ventricle if the RV is exposed to excessive pressure and/or volume loading. However, in the present study, the majority of patients had normal RV size and normal peak systolic pulmonary artery pressure, indicating that the elevated BNP levels were primarily due to synthesized BNP in both infarct and non-infarct sites in the left ventricle. Therefore, our data indicates that process of RV myocardial dysfunction following MI may be governed by neurohormonal activation which causing ventricular remodeling [27] response to altered LV wall stress rather than increased RV afterload.

RV function provides strong prognostic information in patients with MI [2,28]. Antoni et al. demonstrated that indices of RV function including RV strain were strong predictors of the composite end point all-cause mortality, reinfarction, and hospitalization for heart failure in patients with acute MI treated with primary percutaneous coronary intervention [28]. Similary, reduced RV strain is strongly associated with poor cardiovascular outcomes in patients with OMI in the present study although conventional indices of RV function including RV FAC and TAPSE did not reach statistical significance probably because of small sample size.

### Study limitations

Limitations of this study include the small sample size and retrospective nature of data collection. For example, although no significant relationships between RV an LV longitudinal functions both in patients with BNP <100 pg/ml and patient with 100 <BNP <500 pg/ml were observed, these results can be due to type 2 error. Furthermore, there was a heterogeneous distribution of RV function in our study population. Invasive pressure measurements were not used in this study. Therefore, peak systolic pulmonary artery pressure and PVR estimated by using Doppler echocardiography were recruited as markers of the severity of RV pressure overload. However, Doppler-derived estimations of these hemodynamic indices are widely recognized to work well with simultaneous catheter-derived measurements and are widely used clinically. Quantitative assessment of myocardial infarction size was not involved in the present study. Although we assessed the potential impact of residual total occlusion in the culprit lesion responsible for MI on RV function, the presence and severity of stress-induced myocardial ischemia was not evaluated in our study. Finally, three dimensional myocardial tracking in both the left and right ventricle would be warranted for the precise understanding of the pathophysiological mechanism responsible for RV functional impairments after MI.

## Conclusion

Speckle-tracking echocardiography effectively quantified and characterized the RV systolic function and its association with the LV myocardial impairment in patients with OMI. Our data showed that longitudinal RV systolic strain depends highly on longitudinal LV systolic strain, especially in patients having high plasma BNP levels. These results may indicate that longitudinal RV dysfunction following MI does not owe fully to excessive afterload but also due to neurohormonal activation which causing ventricular remodeling response to altered LV wall stress. Application of speckle-tracking echocardiography to conventional measurements of ventricular function could provide a more thorough and quantitative pathophysiological characterization of functional RV adaptation following MI.

## Abbreviations

RV: Right ventricular; MI: Myocardial infarction; PSS: Peak systolic strain; LV: Left ventricular; BNP: Brain natriuretic peptide; E/A: Ratio of peak early to late diastolic transmitral flow velocity; DT: Deceleration time; Ea: Peak early diastolic mitral annular velocity; E/Ea: Ratio of peak early diastolic transmitral flow velocity to Ea; EDA: End-diastolic area; ESA: End-systolic area; FAC: Fractional area change; TAPSE: Tricuspid annular plane systolic excursion; MPI: Myocardial performance index; PA: Pulmonary artery; PVR: Pulmonary vascular resistance; NYHA: New York heart association; HF: Heart failure.

## Competing interests

The authors declare that they have no competing interests.

## Authors’ contributions

KK; Acquisition of data, analysis and interpretation of data, drafting of the manuscript, KD; Corresponding author, protocol design, drafting of the manuscript, critical revision of the manuscript for important intellectual content. MT, YS, KW, and ES: Acquisition and analysis of data, NK, SN, HN, and TY: Interpretation of data, KO and MN: Critical revision of the manuscript for important intellectual content, study supervision, TN and MI: Final approval of the manuscript submitted. All authors read and approved the final manuscript.

## Supplementary Material

Additional file 1 **Movie 1.** Left ventricular (LV) longitudinal strain image in the apical four-chamber view from the same normal subject shown in the Figure 4A. Click here for file

Additional file 2 **Movie 2.** Right ventricular (RV) longitudinal strain image in the apical four-chamber view from the same normal subject shown in the Figure 5A. Click here for file

Additional file 3 **Movie 3.** Left ventricular (LV) longitudinal strain image in the apical four-chamber view from the same patient with old myocardial infarction shown in the Figure 4B. Click here for file

Additional file 4 **Movie 4.** Right ventricular (RV) longitudinal strain image in the apical four-chamber view from the same normal subject shown in the Figure 5B. Click here for file

Additional file 5 **Movie 5.** Left ventricular (LV) longitudinal strain image in the apical four-chamber view from the same patient with old myocardial infarction shown in the Figure 4C. Click here for file

Additional file 6 **Movie 6.** Right ventricular (RV) longitudinal strain image in the apical four-chamber view from the same patient with old myocardial infarction shown in the Figure 5C. Click here for file

## References

[B1] Patten RD Aronovitz MJ Deras-Mejia L Pandian NG Hanak GG Smith JJ Mendelsohn ME Konstam MA Ventricular remodeling in a mouse model of myocardial infarction J Am Coll Cardiol 1998 274 H1812 1820 10.1152/ajpheart.1998.274.5.H1812 9612394

[B2] Zornoff LA Skali H Pfeffer MA St John Sutton M Rouleau JL Lamas GA Plappert T Rouleau JR Moyé LA Lewis SJ Braunwald E Solomon SD Right ventricular dysfunction and risk of heart failure and mortality after myocardial infarction J Am Coll Cardiol 2002 39 1450 1455 10.1016/S0735-1097(02)01804-1 11985906

[B3] Marmor A Geltman EM Biello DR Sobel BE Siegel BA Roberts R Functional response of the right ventricle to myocardial infarction: dependence of the site of left ventricular infarction Circulation 1981 64 1005 1011 10.1161/01.CIR.64.5.1005 7285289

[B4] Rigolin VH Robiolio PA Wilson JS Harrison JK Bashore TM The forgotten chamber: The importance of the right ventricle Catheter Cardiovasc Diag 1995 35 18 28 10.1002/ccd.1810350105 7614536

[B5] Sugiura E Dohi K Onishi K Takamura T Tsuji A Ota S Yamada N Nakamura M Nobori T Ito M Reversible right ventricular regional non-uniformity quantified by speckle-tracking strain imaging in patients with acute pulmonary thromboembolism J Am Soc Echocardiogr 2009 22 1353 1359 10.1016/j.echo.2009.09.005 19836202

[B6] Takamura T Dohi K Onishi K Tanabe M Sugiura E Nakajima H Ichikawa K Nakamura M Nobori T Ito M Left ventricular contraction-relaxation coupling in normal, hypertrophic, and failing myocardium quantified by speckle-tracking global strain and strain rate imaging J Am Soc Echocardiogr 2010 23 747 754 10.1016/j.echo.2010.04.005 20434880

[B7] Nishimura RA Tajik AJ Evaluation of diastolic filling of left ventricle in health and disease: Doppler echocardiography is the clinician's rosetta stone J Am Coll Cardiol 1997 30 8 18 10.1016/S0735-1097(97)00144-7 9207615

[B8] Nagueh SF Appleton CP Gillebert TC Marino PN Oh JK Smiseth OA Waggoner AD Flachskampf FA Pellikka PA Evangelista A Recommendations for the evaluation of left ventricular diastolic function by echocardiography J Am Soc Echocardiogr 2009 22 107 133 10.1016/j.echo.2008.11.023 19187853

[B9] Rudski LG Lai WW Afilalo J Hua L Handschumacher MD Chandrasekaran K Solomon SD Louie EK Schiller NB Guidelines for the echocardiographic assessment of the right heart in adults: A report from the american society of echocardiography endorsed by the european association of echocardiography, a registered branch of the european society of cardiology, and the canadian society of echocardiography J Am Soc Echocardiogr 2010 23 685 713 10.1016/j.echo.2010.05.010 20620859

[B10] Yeo TC Dujardin KS Tei C Mahoney DW McGoon MD Seward JB Value of a doppler-derived index combining systolic and diastolic time intervals in predicting outcome in primary pulmonary hypertension Am J Cardiol 1998 81 1157 1161 10.1016/S0002-9149(98)00140-4 9605059

[B11] Abbas AE Fortuin FD Schiller NB Appleton CP Moreno CA Lester SJ A simple method for noninvasive estimation of pulmonary vascular resistance J Am Coll Cardiol 2003 41 1021 1027 10.1016/S0735-1097(02)02973-X 12651052

[B12] Mor-Avi V Lang RM Badano LP Belohlavek M Cardim NM Derumeaux G Galderisi M Marwick T Nagueh SF Sengupta PP Sicari R Smiseth OA Smulevitz B Takeuchi M Thomas JD Vannan M Voigt JU Zamorano JL Current and evolving echocardiographic techniques for the quantitative evaluation of cardiac mechanics: ASE/EAE consensus statement on methodology and indications endorsed by the Japanese Society of Echocardiography J Am Soc Echocardiogr 2011 24 277 313 10.1016/j.echo.2011.01.015 21338865

[B13] Dohi K Onishi K Gorcsan J 3rd Lopez-Candales A Takamura T Ota S Yamada N Ito M Role of radial strain and displacement imaging to quantify wall motion dyssynchrony in patients with left ventricular mechanical dyssynchrony and chronic right ventricular pressure overload Am J Cardiol 2008 101 1206 1212 10.1016/j.amjcard.2007.11.077 18394460

[B14] Kakuta Y Okumi M Ichimaru N Abe T Nonomura N Okuyama A Kojima Y Isaka Y Takahara S Imai E Horio M Utility of the Japanese GFR estimation equation for evaluating potential donor kidney function Clin Exp Nephrol 2010 14 63 67 10.1007/s10157-009-0224-0 19806425

[B15] Leather HA Ama R Missant C Rex S Rademakers FE Wouters PF Longitudinal but not circumferential deformation reflects global contractile function in the right ventricle with open pericardium Am J Physiol Heart Circ Physiol 2006 290 H2369 2375 10.1152/ajpheart.01211.2004 16399859

[B16] Brown SB Raina A Katz D Szerlip M Wiegers SE Forfia PR Longitudinal shortening accounts for the majority of right ventricular contraction and improves after pulmonary vasodilator therapy in normal subjects and patients with pulmonary arterial hypertension Chest 2011 140 27 33 10.1378/chest.10-1136 21106653

[B17] Alpendurada F Guha K Sharma R Ismail TF Clifford A Banya W Mohiaddin RH Pennell DJ Cowie MR McDonagh T Prasad SK Right ventricular dysfunction is a predictor of non-response and clinical outcome following cardiac resynchronization therapy J Cardiovasc Magn Reson 2011 13 68 10.1186/1532-429X-13-68 22040270 PMC3217913

[B18] de Groote P Fertin M Goeminne C Petyt G Peyrot S Foucher-Hossein C Mouquet F Bauters C Lamblin N Right ventricular systolic function for risk stratification in patients with stable left ventricular systolic dysfunction: Comparison of radionuclide angiography to echodoppler parameters Eur Heart J 2012 33 2672 2679 10.1093/eurheartj/ehs080 22453651

[B19] Haddad F Hunt SA Rosenthal DN Murphy DJ Right ventricular function in cardiovascular disease, part i: Anatomy, physiology, aging, and functional assessment of the right ventricle Circulation 2008 117 1436 1448 10.1161/CIRCULATIONAHA.107.653576 18347220

[B20] Toldo S Bogaard HJ Van Tassell BW Mezzaroma E Seropian IM Robati R Salloum FN Voelkel NF Abbate A Right ventricular dysfunction following acute myocardial infarction in the absence of pulmonary hypertension in the mouse PLoS One 2011 6 e18102 10.1371/journal.pone.0018102 21455304 PMC3063789

[B21] Alam M Wardell J Andersson E Samad BA Nordlander R Right ventricular function in patients with first inferior myocardial infarction: Assessment by tricuspid annular motion and tricuspid annular velocity Am Heart J 2000 139 710 715 10740156 10.1016/s0002-8703(00)90053-x

[B22] Buckberg G Athanasuleas C Saleh S Septal myocardial protection during cardiac surgery for prevention of right ventricular dysfunction Anadolu Kardiyol Derg 2008 8 Suppl 2 108 116 19028643

[B23] Quaife RA Christian PE Gilbert EM Datz FL Volkman K Bristow MR Effects of carvedilol on right ventricular function in chronic heart failure Am J Cardiol 1998 81 247 250 10.1016/S0002-9149(97)00874-6 9591916

[B24] Silver MA Maisel A Yancy CW McCullough PA Burnett JC Jr Francis GS Mehra MR Peacock WF 4th Fonarow G Gibler WB Morrow DA Hollander J Bnp consensus panel 2004: A clinical approach for the diagnostic, prognostic, screening, treatment monitoring, and therapeutic roles of natriuretic peptides in cardiovascular diseases Congest Heart Fail 2004 10 1 30 15604859 10.1111/j.1527-5299.2004.03271.x

[B25] Shah MR Hasselblad V Tasissa G Christenson RH Binanay C O'Connor CM Ohman EM Stevenson LW Califf RM Rapid assay brain natriuretic peptide and troponin i in patients hospitalized with decompensated heart failure (from the evaluation study of congestive heart failure and pulmonary artery catheterization effectiveness trial) Am J Cardiol 2007 100 1427 1433 10.1016/j.amjcard.2007.06.035 17950802

[B26] Vogelsang TW Jensen RJ Monrad AL Russ K Olesen UH Hesse B Kjaer A Independent effects of both right and left ventricular function on plasma brain natriuretic peptide Eur J Heart Fail 2007 9 892 896 10.1016/j.ejheart.2007.05.015 17613272

[B27] Santamore WP Dell'Italia LJ Ventricular interdependence: Significant left ventricular contributions to right ventricular systolic function Prog Cardiovasc Dis 1998 40 289 308 10.1016/S0033-0620(98)80049-2 9449956

[B28] Antoni ML Scherptong RW Atary JZ Boersma E Holman ER van der Wall EE Schalij MJ Bax JJ Prognostic value of right ventricular function in patients after acute myocardial infarction treated with primary percutaneous coronary intervention Circ Cardiovasc Imaging 2010 3 264 271 10.1161/CIRCIMAGING.109.914366 20190280

